# Association between hyperuricemia and kidney stones in Southern China: a multicentre cross-sectional study

**DOI:** 10.3389/fendo.2026.1611287

**Published:** 2026-01-23

**Authors:** Yuwen Zhong, Rongxin He, Ganglin Kang, Zhongfang Zhou, Kaimin Xiao, Li Li

**Affiliations:** 1 Department of Pathology, Luzhou Maternal and Child Health Hospital (Luzhou Second People’s Hospital), Luzhou, China; 2 Department of Neurology, The Affiliated Minzu Hospital of Guangxi Medical University, Nanning, China; 3 Health Management Center, The Affiliated Traditional Chinese Medicine Hospital, Southwest Medical University, Luzhou, China; 4 Department of Neurology, People’s Hospital of Ganxian District, Ganzhou, China

**Keywords:** hyperuricemic, kidney stone, multicenter study, multicentre cross-sectional study, non-linear relationship

## Abstract

**Background:**

Hyperuricemia has been identified as a significant independent risk factor for kidney stones. However, a paucity of research has been conducted on the correlation between hyperuricemia in the general population and the prevalence of kidney stones. Southern China has a high incidence of kidney stones, and analysis of data from health check-ups can help to identify those at risk of developing kidney stones. This is of positive clinical significance for the prevention of kidney stones in hyperuricemia populations.

**Methods:**

A multicentre cross-sectional study was conducted using data from medical examination centres in four hospitals located in three southern Chinese provinces from 2022 to 2024. The analysis employed a combination of statistical methods, including logistic regression to identify independent risk factors for kidney stones in individuals with hyperuricemia. Additionally, a restricted cubic spline (RCS) method was utilised to examine the dose-response relationship between age, BMI, and serum uric acid levels and the risk of kidney stones. The study also employed a threshold effect analysis to identify the threshold inflection point between age and the risk of kidney stones.

**Results:**

The total health data of 2739 medical examiners were included in this study. The prevalence of kidney stones was found to be 25.48% (1.28% in females and 24.21% in males) in the hyperuricemia population. The application of logistic regression revealed that age, BMI, serum uric acid, sex, urine leukocyte abnormality, and urine erythrocyte abnormality functioned as independent risk factors, while water intake was identified as a protective factor. Furthermore, the results of the RCS indicated a nonlinear relationship between age and the prevalence of renal stones (P nonlinear < 0.001). Threshold effect results showed that for individuals under the age of 44, the risk of developing kidney stones increased by 6.3% with each additional year of age (P < 0.05).

**Conclusion:**

In the hyperuricemic population, age, BMI, serum annual acid, sex, abnormal leukocytes in urine and abnormal red blood cells in urine were identified as independent risk factors for developing kidney stones, while water intake was found to be a protective factor. The relationship between age and the development of kidney stones in hyperuricemia is non-linear.

## Introduction

Hyperuricemia (HUA) is a condition in which blood uric acid is elevated due to decreased uric acid excretion or impaired purine metabolism. Epidemiological studies have shown that the incidence of hyperuricemia is increasing year by year worldwide, and the incidence of hyperuricemia in China is also increasing year by year, and there is a tendency of youthfulness (1, 2). Hyperuricemia has become one of the metabolic diseases that threaten human health.

Kidney stones are one of the most common diseases in urology and their incidence varies by region, with a global prevalence of approximately 1-20% in each country or region (3). The prevalence is higher in men than in women, with a five-year recurrence rate of up to 50% (4). Some studies have shown that kidney stones are closely associated with diseases such as obesity, diabetes mellitus, hypertension and metabolic syndrome, which severely impair organ function (5–7). With the changes in human dietary structure, lifestyle and other modalities, the incidence of kidney stones will continue to increase and pose a serious threat to human health.

Hyperuricaemia is an independent risk factor for kidney stones (8). Not only does hyperuricaemia increase the risk of developing uric acid stones, it also promotes the formation of calcium oxalate stones and mixed stones (9, 10). Studies have shown that elevated serum uric acid levels can increase uric acid excretion in urine, thereby lowering urine pH and promoting uric acid crystallisation (11). This also facilitates calcium oxalate crystallisation through heterogeneous nucleation (12). In the renal tubules, high concentrations of uric acid can cause uric acid crystal precipitation and stimulate inflammatory responses in renal tubular epithelial cells. This leads to the release of inflammatory factors and results in renal tubular interstitial inflammation and fibrosis. This, in turn, causes reabsorption disorder and further promotes uric acid crystal deposition and stone formation (13, 14). A large cohort study conducted in South Korea demonstrated that elevated serum uric acid levels in men are independently associated with an increased risk of kidney stones. The study also suggested that men with hyperuricemia have at least an 11% higher risk of developing kidney stones than those with normal uric acid levels (15). Researchers from China discovered that an increase of 100 μmol/L in uric acid levels in men suffering from hyperuricaemia was associated with a minimum 9.2% increase in the risk of developing kidney stones, in comparison to men with low serum uric acid levels (16). A single-centre study found that, of Chinese adults with recurrent kidney stones, 52.4% had hyperuricaemia (17).

With the continuous development of society and improvement of economic level, the prevalence of various metabolic diseases is increasing, and the prevalence of hyperuricemia with kidney stones varies in different regions and populations due to the influence of factors such as different regions and populations (2, 3). Therefore, the present study was conducted to investigate the prevalence and influencing factors of kidney stones in individuals aged 18–80 years with hyperuricemia in the medical examination centres of multiple provinces in southern China by collecting data from these centres, to identify high-risk groups, and to provide a basis for the prevention and treatment of hyperuricemia-associated kidney stones.

## Methods

### Study population and design

**Figure 1:**
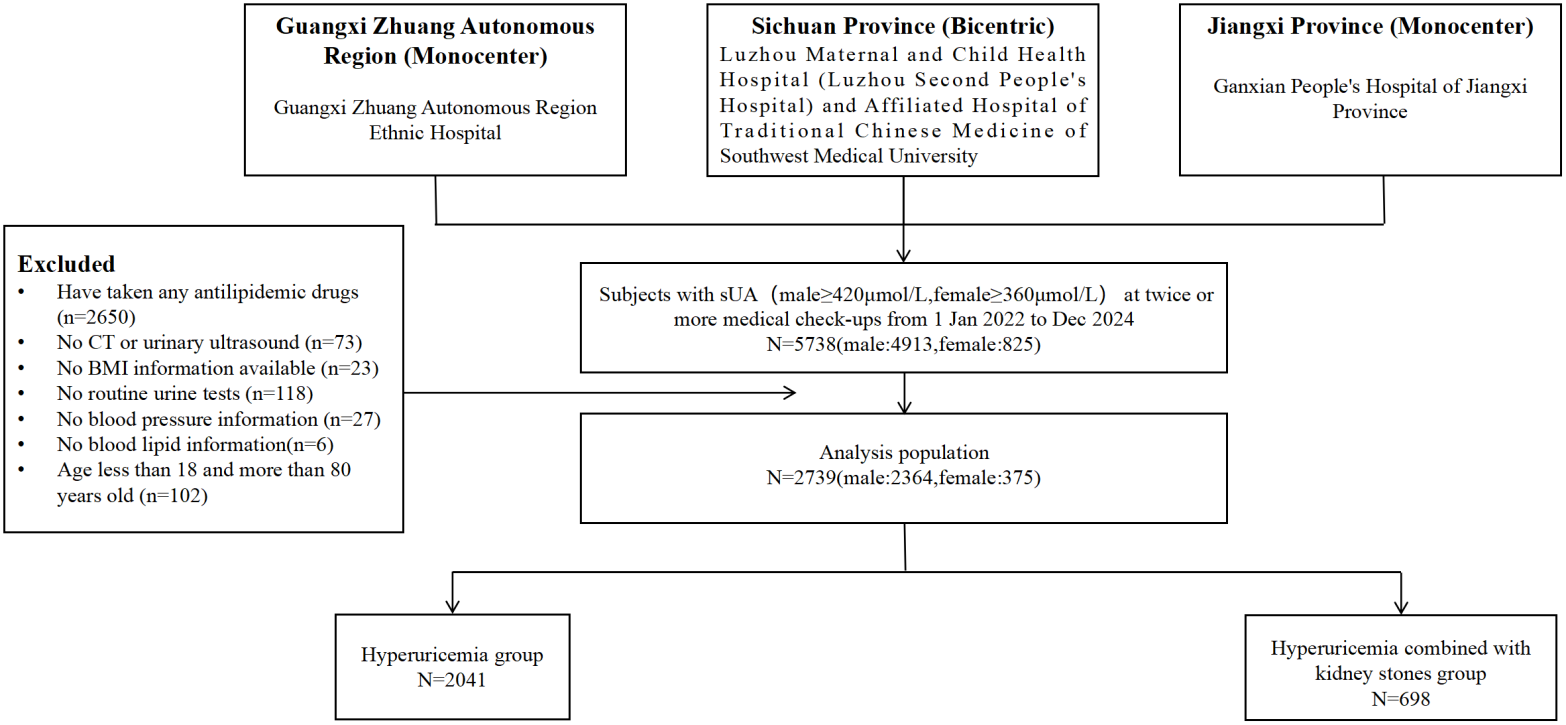
Flow chart of the study design.

This is a cross-sectional study by collecting data from the hyperuricemic population who underwent health check-ups at the physical examination centres of the Luzhou Maternal and Child Health Hospital (Luzhou Second People’s Hospital), Sichuan Province, the Traditional Chinese Medicine Hospital of Southwest Medical University, the Ethnic Hospital of Guangxi Zhuang Autonomous Region, and the People’s Hospital of Ganxian County, Ganzhou City, Jiangxi Province during the period 2022-01–01 to 2024-12-30. After data cleaning, the study included 2,739 participants who met the criteria for natriuresis. The specific screening process was detailed in the flowchart (**Figure 1**).

### Ethics

The study was approved by the medical ethics committees of each center separately, and the study protocol followed the ethical principles of the Declaration of Helsinki and its subsequent amendments. Signed informed consent was obtained from all participants in the questionnaire section of this study. We ensured the protection of participants’ rights and privacy, and all data were anonymised according to ethical standards. The remaining data were obtained from the hospital’s electronic medical record system and were anonymised during the study to ensure patient privacy. The study was conducted in strict accordance with the Enhanced Reporting of Observational Studies in Epidemiology guidelines (18).

### Variable

The data information consisted mainly of questionnaires and routine health examination indicators. The questionnaire included whether participants took uricate-lowering medications, whether they had a history of chronic kidney disease, serious underlying diseases or malignant tumours, whether they had a history of thyroid disease or diseases that cause hypercalcaemia, and the participants’ lifestyle habits such as diet and exercise. Physical examination indicators included sex, age, BMI, blood pressure, blood glucose, kidney function, urinary frequency, and imaging data such as abdominal ultrasound or abdominal CT.

### Diagnosis of related diseases

Hyperuricemia: patients with two consecutive serum uric acids consistent with a diagnosis of hyperuricemia or who have been diagnosed with hyperuricemia (19). Kidney stones: CT or urinary ultrasound reveals kidney stones (8). Diabetes mellitus: questionnaire asked ‘Do you take hypoglycemic medication or have you been told by your doctor that you have diabetes mellitus?’ (20). Hypertension: questionnaire asking ‘Have you been told by your doctor that you have high blood pressure’ or systolic blood pressure ≥ 140 mmHg and/or diastolic blood pressure ≥ 90 mmHg measured at rest (21). Hyperlipidemia was defined as respondents reporting that they were taking lipid-lowering drugs, had been informed by their doctors that they had hyperlipidemia, or had total cholesterol ≥ 5.2 mmol/L, triglyceride ≥ 1.7 mmol/L, LDL-C ≥ 3.4 mmol/L, or HDL-C < 1.0 mmol/L. Meeting any of these criteria was considered diagnostic for hyperlipidemia (22).

### Statistical analysis

Data analysis was performed using SPSS 26.0 software. Continuous variables with normal distribution were presented as mean ± standard deviation (SD), and comparisons between two groups were made using the independent samples t-test. Continuous variables without normal distribution were presented as median with interquartile range (IQR), and comparisons between two groups were made using the Mann-Whitney U test. Categorical variables were presented as number (%), and comparisons between two groups were made using the chi-squared test. Multivariate logistic regression analysis was used to investigate risk factors for kidney stones in patients with hyperuricemia. Multicollinearity among the independent variables in the final multivariable logistic regression model was assessed using the variance inflation factor (VIF). Spearman correlation analysis was used to screen for correlations between risk factors. Restricted cubic spline analysis was used to analyze the linear and nonlinear relationships between age, BMI, and uric acid in specific populations. Curve fitting and threshold effect analysis were used to analyze the dose-response relationship between age and kidney stones. Sensitivity analyses were conducted to assess the robustness of the primary findings. A significance level of α = 0.05 was used for all tests.

## Results

### Baseline data on the study population

In this cross-sectional study, a total of 2,739 hyperuricemic patients with available physical examination data were included and divided into two groups (stone group and non-stone group) based on the diagnostic criteria for kidney stones for statistical analysis. As shown in **Table 1**, the mean serum uric acid level was significantly higher in the hyperuricemia with stones group (497 µmol/L) compared to the pure hyperuricemia group (476 µmol/L). Additionally, the hyperuricemia with stones group had a higher mean age (46 years) than the pure hyperuricemia group (39 years). The prevalence of kidney stones among hyperuricemic patients was 25.48%, with a rate of 1.28% in women and 24.21% in men. Among the entire study population, we found a prevalence of moderate-to-severe CKD at 2.08%. Specifically, among individuals with kidney stone disease, the prevalence of severe CKD or higher reached 4.01%. Further analysis indicated that age, sex, BMI, diabetes mellitus, hypertension (both systolic and diastolic), serum uric acid, creatinine, triglycerides, high-density lipoprotein (HDL), urinary leukocytes, urinary erythrocytes, urinary proteins, urinary pH, urinary epithelial cells, urinary crystals, eGFR, CKD and daily water intake were all significantly associated with the development of kidney stones in patients with hyperuricemia (P < 0.05).

**Table 1:** Baseline characteristics of patients with hyperuricemia and kidney stones in a multicenter study in Southern China.

Variable	Non_KSD_Group (2041)	KSD_Group (698)	P value
Age (years)	39.00 (33.00,52.00)	46.00 (35.00,54.00)	<0.001
Sex			<0.001
Male	1701 (83.34)	663 (94.99)	
Female	340 (16.66)	35 (5.01)	
BMI (kg/m^2^)	25.75 (23.61,27.91)	26.58 (24.72,28.95)	<0.001
Diabetes mellitus			<0.001
Yes	263 (12.89)	139 (19.91)	
No	1778 (87.11)	559 (80.09)	
Hypertension			<0.001
Yes	691 (33.86)	338 (48.42)	
No	1350 (66.14)	360 (51.58)	
Systolic blood (mmHg)	131.00 (120.00,140.00)	136.00 (126.00,147.00)	<0.001
Diastolic blood (mmHg),	84.00 (76.00,91.00)	88.00 (80.00,96.00)	<0.001
Serum uric acid (µmol/L)	476.00 (442.00,522.00)	497.00 (458.00,546.00)	<0.001
Serum creatinine (µmol/L)	83.00 (73.00,91.00)	87.00 (77.00,95.00)	<0.001
Serum urea nitrogen (mmol/L)	4.88 (4.15,5.72)	4.92 (4.13,5.87)	0.526
Total cholesterol (mmol/L)	5.15 (4.50,5.79)	5.21 (4.59,5.88)	0.147
Triglyceride (mmol/L)	2.05 (1.37,3.10)	2.33 (1.47,3.46)	<0.001
Low density lipoprotein (mmol/L)	3.05 (2.54,3.59)	3.09 (2.57,3.57)	0.489
High density lipoprotein (mmol/L)	1.20 (1.05,1.38)	1.18 (1.03,1.35)	0.027
Hyperlipemia			0.215
Yes	1598 (78.30)	562 (80.52)	
No	443 (21.70)	136 (19.48)	
WBC			<0.001
Normal	1705 (83.54)	521 (74.64)	
Abnormal	336 (16.46)	177 (25.36)	
WRC			<0.001
Normal	1674 (82.02)	502 (71.92)	
Abnormal	367 (17.98)	196 (28.08)	
Urinary protein			0.005
Negative	1886 (92.41)	621 (88.97)	
Positive	155 (7.59)	77 (11.03)	
Urine pH			0.021
<6.0	786 (38.51)	303 (43.41)	
≥6	1255 (61.49)	395 (56.59)	
Urine epithelial cells			<0.001
Negative	1849 (90.59)	672 (96.28)	
Positive	192 (9.41)	26 (3.72)	
Urine crystal			<0.001
Negative	1793 (87.85)	578 (82.81)	
Positive	248 (12.15)	120 (17.19)	
Smoking			0.871
Yes	653 (32.00)	221 (31.66)	
No	1388 (68.00)	477 (68.34)	
Alcohol			0.662
Yes	1186 (58.11)	399 (57.16)	
No	855 (41.89)	299 (42.84)	
Water intake			0.005
Yes	614 (30.08)	171 (24.50)	
No	1427 (69.92)	527 (75.50)	
High-purine diet			0.129
Yes	1219 (59.73)	394 (56.45)	
No	822 (40.27)	304 (43.55)	
Physical exercise			0.224
Yes	1278 (62.62)	419 (60.03)	
No	763 (37.38)	279 (39.97)	
eGFR (mL/min/1.73 m²)	99.88 ± 16.26	95.42 ± 17.25	<0.001
Chronic Kidney Disease			<0.001
G1	1548 (75.85%)	460 (65.90%)	
G2	464 (22.73%)	210 (30.09%)	
G3	24 (1.18%)	24 (3.44%)	
G4	5 (0.24%)	4 (0.57%)	

WBC, the number of white blood cells in the urine; WRC, the number of red blood cells in the urine; KSD_Group, kidney stone disease group; Non_KSD_Group, without kidney stone disease group; An abnormal number of leukocytes in urine is defined as ≥ 5/HP; an abnormal number of erythrocytes in urine as ≥ 3/HP; abnormal epithelial cells in urine as a normal squamous epithelial cell count > 5 or a normal non-squamous epithelial cell count > 28.

Smoking: According to the World Health Organization’s criteria, a smoker is defined as a person who has smoked for 6 consecutive or cumulative months or more in their lifetime.

Alcohol: Alcohol intake in the adult population ranges from 0 to 1.87 standardized cups per day (1 standardized cup is equivalent to 10 g of pure ethanol, which is approximately 100 mL red wine at 13% alcohol by volume or 1 can of 375 mL beer at 3.5% alcohol by volume).

Water intake: Drinking more than 2,000 mL water per day is advised.

High-purine diet: A high-purine diet is defined as more than 150 mg high-purine foods per 100 g food.

Physical exercise: Accumulating 150 minutes or more of moderate-intensity exercise per week is recommended.

eGFR (mL/min/1.73 m²): We calculated eGFR using the CKD-EPI creatinine formula recommended by the 2021 Kidney Disease.

Chronic Kidney Disease: Classified according to the 2024 KDIGO Chronic Kidney Disease Guidelines.

### Logistics regression analysis

In this study, multifactorial logistic regression analysis was conducted to identify independent risk factors for kidney stone formation in hyperuricemic patients. As shown in **Table 2**, age, BMI, serum uric acid, male, abnormal urinary leukocytes, and abnormal urinary erythrocytes were identified as independent risk factors for kidney stone formation in this population. Additionally, achieving adequate daily water intake was found to be a protective factor against kidney stone formation in patients with hyperuricemia. We calculated the VIF for all seven independent variables in the final model to evaluate potential multicollinearity. All VIF values were significantly lower than the traditional critical value of 5 (range 1.005-1.241, see **Supplementary Table 1S**), indicating that the model estimation results are reliable.

**Table 2:** Univariate and multivariable binary logistic regression analysis for hyperuricemia with kidney stone.

Variables	Univariate			Multivariable		
	OR	95%CI	P-value	OR	95%CI	P-value
Age	1.018	1.011-1.025	<0.001	1.016	1.008-1.025	**<0.001**
BMI(kg/m^2^)	1.086	1.059-1.113	<0.001	1.065	1.035-1.097	**<0.001**
Sex(male/female)	3.786	2.643-5.423	<0.001	3.089	1.963-4.862	**<0.001**
Diabetes mellitus(+/-)	1.681	1.340-2.108	<0.001	1.208	0.934-1.561	0.150
Hypertension(+/-)	1.834	1.541-2.184	<0.001	1.063	0.792-1.426	0.684
Systolic BP	1.019	1.014-1.024	<0.001	1.001	0.991-1.010	0.977
Diastolic BP	1.030	1.022-1.037	<0.001	1.011	0.997-1.024	0.116
Uric acid	1.005	1.003-1.006	<0.001	1.003	1.001-1.004	**<0.001**
Serum creatinine	1.016	1.011-1.021	<0.001	1.004	0.998-1.010	0.187
Blood urea nitrogen	1.014	0.969-1.061	0.549			
TC	1.062	0.980-1.152	0.144			
TG	1.033	1.009-1.058	0.007	1.010	0.984-1.037	0.446
LDL	1.023	0.916-1.142	0.691			
HDL	0.818	0.606-1.105	0.190			
Urine epithelial cells(+/-)	0.373	0.245-0.567	<0.001	0.647	0.391-1.069	0.089
Urine crystals(+/-)	1.501	1.184-1.903	<0.001	1.260	0.977-1.626	0.075
Urine protein(+/-)	1.509	1.131-2.012	0.005	0.792	0.571-1.099	0.162
Urine WBC counts(+/-)	1.724	1.402-2.120	<0.001	1.708	1.345-2.169	**<0.001**
Urine RBC counts (+/-)	1.781	1.458- 2.175	<0.001	1.568	1.252-1.964	**<0.001**
Urine pH(<6/≥6)	1.225	1.029-1.458	0.023	1.140	0.946-1.374	0.169
Hyperlipidemia(+/-)	1.146	0.924-1.420	0.215			
Water intake(Yes/No)	0.754	0.619-0.918	0.005	0.766	0.623-0.941	**0.011**
Physical exercise(Yes/No)	0.897	0.752-1.069	0.224			
High-purine diet(Yes/No)	0.874	0.735-1.040	0.129			
Drinking(Yes/No)	0.962	0.809-1.145	0.662			
Smoking(Yes/No)	0.985	0.819-1.184	0.871			
eGFR	0.984	0.979-0.989	<0.001	0.981	0.956-1.007	0.143
CKD						
G1	1.00 (Reference)			1.00 (Reference)		
G2	1.523	1.255-1.848	<0.001	1.147	0.826-1.593	0.411
G3	3.365	1.893-5.982	<0.001	2.615	1.037-6.595	**0.042**
G4	2.692	0.720-10.067	0.141	2.707	0.475-15.442	0.262
Kidney failure(Yes/No)	0.345	0.204 ~ 0.584	<0.001	0.366	0.064- 2.084	0.257

Age, male, serum uric acid, BMI,urinary erythrocyte abnormalities, and urinary leukocyte abnormalities are independent risk factors for kidney stone formation in hyperuricemia. Drinking water is a protective factor in the risk of kidney stones with hyperuricemia. OR, odds ratio; CI, confidence interval. Bold values denote statistical significance (P < 0.05).

### Correlation analysis

**Figure 2:**
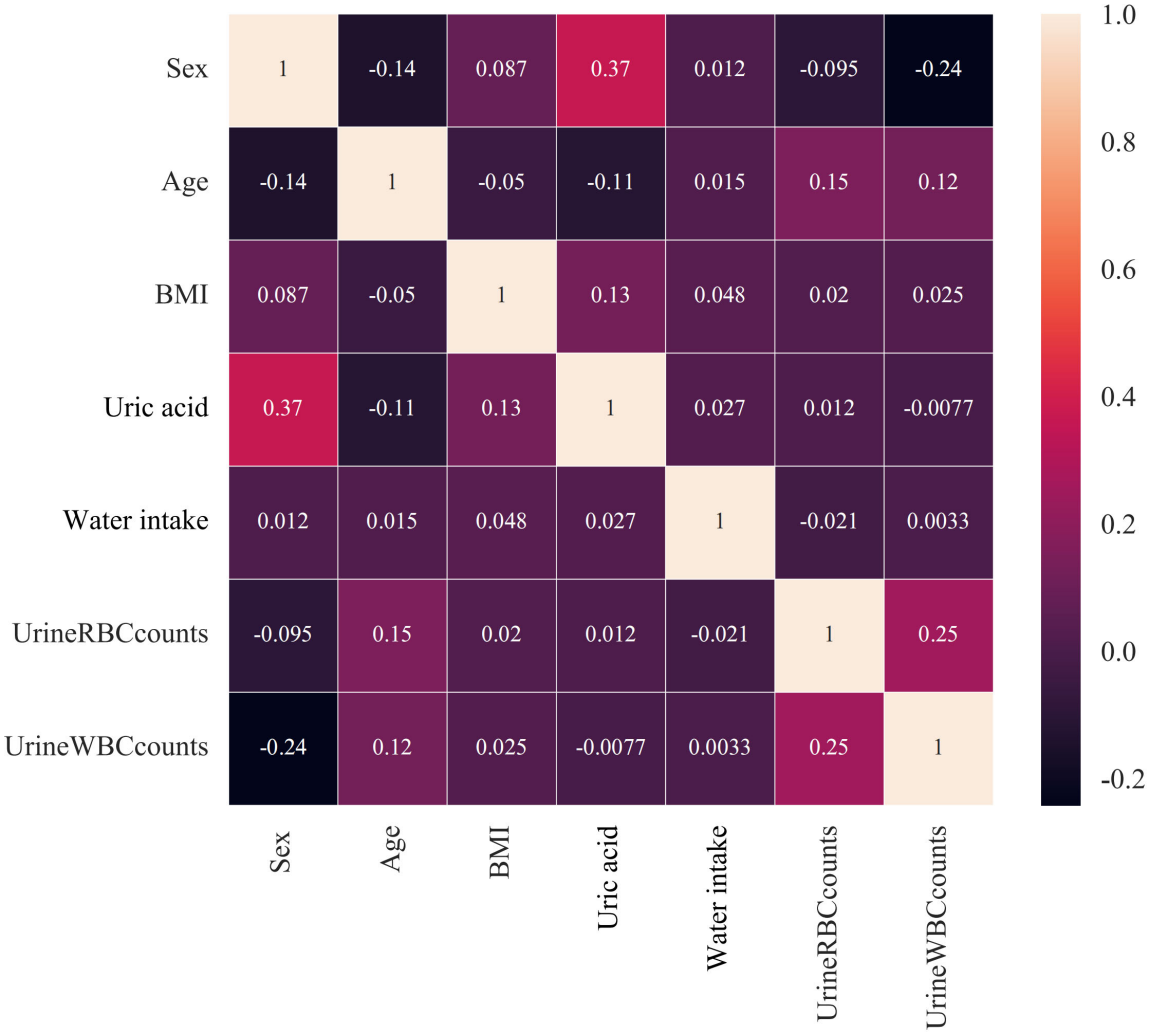
Correlation analysis among independent risk factors. No significant strong correlation was found among the independent risk factors.

Spearman correlation analysis was used to screen for associations between independent risk factors. **Figure 2** shows that no strong correlation was found between the independent risk factors.

### Dose-response relationship between age, BMI, serum uric acid and risk of kidney stone prevalence

**Figure 3:**
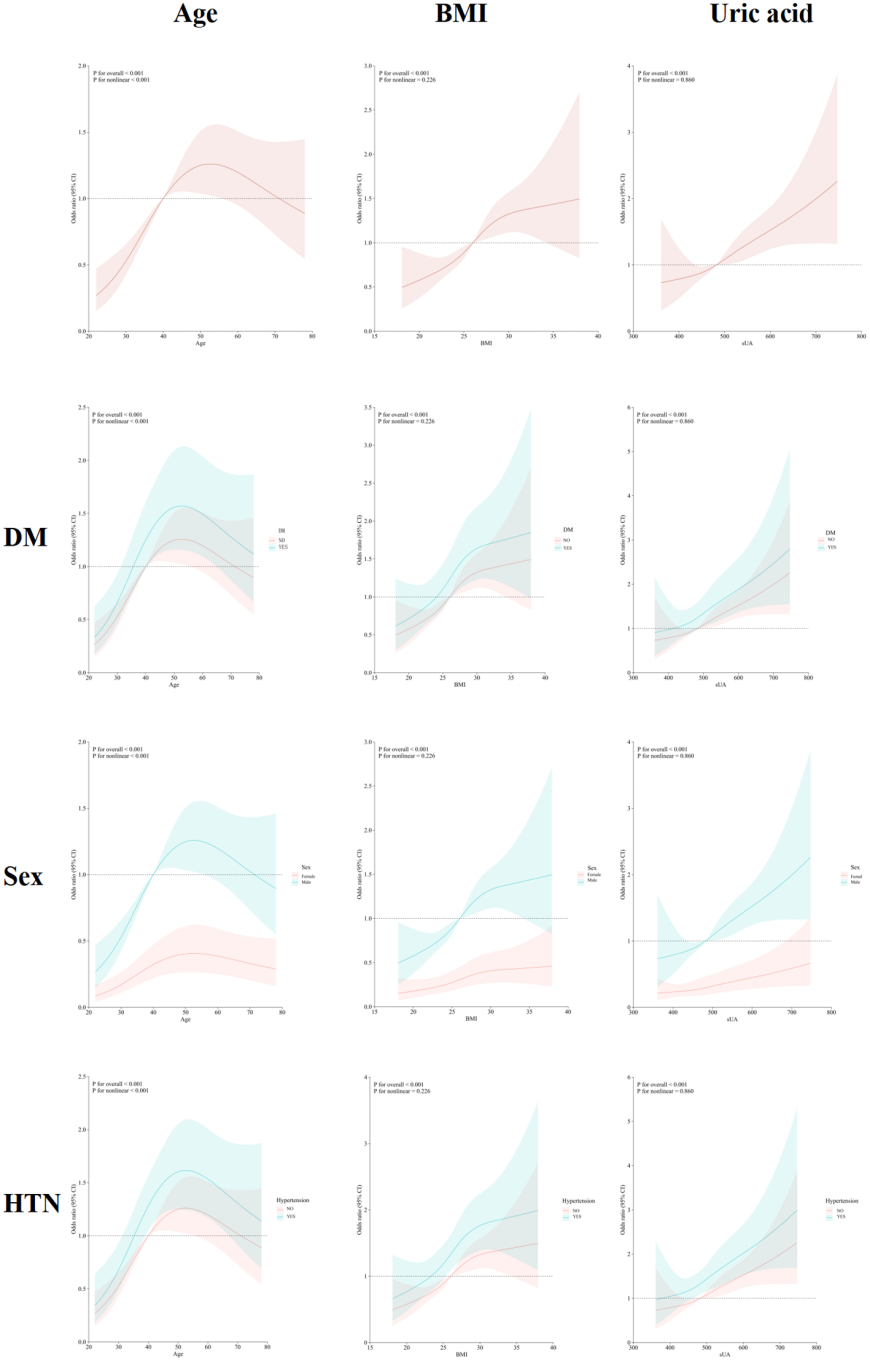
Dose-response relationship between age, BMI, serum uric acid and risk of kidney stone prevalence. Following adjustment for confounding factors including sex, age, BMI, diabetes mellitus, hypertension, hyperlipidaemia, daily water intake and high-purine diet, a nonlinear relationship between age and the prevalence of kidney stones was identified.

As shown in **Figure 3**, after adjusting for sex, age, BMI, diabetes mellitus, hypertension, hyperlipidaemia, daily water intake, and high-purine dietary factors, RCS analysis was used to examine the relationship between the three independent risk factors (age, BMI, and serum uric acid) and the risk of kidney stone development in hyperuricemic patients. A non-linear relationship was observed between age and kidney stone risk (P for overall < 0.001; P for non-linear < 0.001), with age being an independent risk factor for kidney stone development between 40 and 70 years. No significant non-linear relationships were found between BMI (P for overall < 0.001; P for non-linear = 0.226) or serum uric acid (P for overall < 0.001; P for non-linear = 0.860) and kidney stone risk. However, when BMI exceeded 26.01 kg/m² or serum uric acid levels exceeded 482 µmol/L, the odds ratio (OR) for kidney stone development was greater than 1. RCS analysis further revealed that patients with diabetes mellitus or hypertension had lower thresholds for these risk factors compared to those without these conditions, and men had a higher risk of kidney stone development than women.

### Curve fitting and threshold effect analysis of age and risk of kidney stone occurrence

In this study, among all continuous variables of independent risk factors, only age exhibited a non-linear relationship with the risk of kidney stone development. To further explore this relationship, curve fitting and threshold effect analyses were conducted, with adjustments made for sex, BMI, diabetes, hypertension, hyperlipidaemia, daily water intake, and high-purine dietary factors. The curve fitting results confirmed a non-linear association between age and kidney stone risk, aligning with the RCS findings (Detailed information is provided in **Supplementary Figure 1**). Additionally, threshold effect analysis (**Table 3**) revealed that in individuals younger than 44 years, the risk of kidney stone development increased by 6.3% per additional year of age.

**Table 3:** Analysis of threshold effects of age on kidney stone risk.

Kidney stone	Adjust OR(95%CI)	P-value
Fitting by the standard linear modeI	1.019 (1.011, 1.027)	<0.001
Fitting by the two-piecewise linear modeII		
Inflection point	44	
Age < 44	1.063 (1.043, 1.084)	<0.001
Age > 44	0.991(0.978, 1.005)	0.214
Log likelihood ratio		<0.001

Adjustment variables: sex, BMI, diabetes mellitus, hypertension, hyperlipidemia, water intake, and High-purine die.

### Sensitivity analysis

To assess whether the impact of drug use on kidney stone formation in hypertensive and diabetic populations causes confounding, we conducted a sensitivity analysis and ruled out potential confounding caused by drugs such as antihypertensive drugs. The **Supplementary Table 2S** results showed that even in the population with both hypertension and diabetes, there were no significant changes in the direction and magnitude of the effect values of uric acid and eGFR compared with the total population. To investigate potential differences in the association between hyperuricemia and kidney stones across populations with varying renal function, we performed a stratified analysis and interaction tests according to the clinical staging criteria of chronic kidney disease. The **Supplementary Table 3S** results from stratified analysis indicated that the interaction between CKD stage and serum uric acid levels was not statistically significant (P for interaction = 0.207), suggesting that the strength of the association between hyperuricemia and kidney stones did not significantly differ across renal function subgroups.

## Discussions

Hyperuricemia is one of the key aetiologies of kidney stones, and its promotion of stone formation may be attributed to factors such as urinary pH alterations, kidney injury, and the presence of metabolic syndrome (6, 23). When serum uric acid levels rise further, urate concentration in the kidneys reaches saturation, leading to crystal precipitation, stone formation, and subsequent renal damage (24). Previous studies have demonstrated a positive correlation between hyperuricemia and kidney stone incidence (25). In this multicentre study, the prevalence of kidney stones among hyperuricemic patients was 25.48%, which is significantly higher than the average prevalence in China (26). Despite the absence of comprehensive medication data in the medical records, this study utilised sensitivity analyses to evaluate the potential confounding effects of medications, including diuretics, to the greatest extent feasible. The findings provide indirect evidence that such medications do not introduce significant bias. Consequently, this study analysed data from a multicentre medical examination population to identify independent risk factors for kidney stone development in hyperuricemic patients, aiming to offer a scientific foundation for the prevention and treatment of kidney stone disease in hyperuricemic patients in southern China.

In this study, the mean ages of participants in the stone group and non-stone group were 46 years and 39 years, respectively. The analysis revealed a nonlinear relationship between age and kidney stone risk in patients with hyperuricemia (P for non-linearity < 0.01). This was determined through curve fitting, threshold effect analysis, and restrictive cubic splines. A threshold effect was also identified: among individuals under 44 years old, each additional year of age increased kidney stone risk by 6.3% (P < 0.001). However, beyond the age of 44 years, no significant association between age and stone risk was observed (P > 0.05), potentially reflecting age-related renal function decline and reduced urinary calcium excretion (27–29). As demonstrated in previous studies, an elevated excretion of urinary factors, including calcium, magnesium, and uric acid, has been shown to promote the development of kidney stones (30–32). Perinpam et al. (27)discovered a negative correlation between age and the excretion of these urinary factors (P < 0.05). Consequently, the likelihood of developing kidney stones may diminish with increasing age. Multifactorial logistic regression identified male, BMI, and high serum uric acid as independent risk factors for kidney stones in hyperuricemia. Specifically, hyperuricemic men had 3 times the stone risk of women (P < 0.001), and each 1-unit BMI increase raised the risk by 5% (P = 0.026). Higher serum uric acid levels were also linked to greater stone risk, aligning with other clinical analyses (15, 33).

Studies have shown that kidney stone disease and urinary tract infections often coexist and share a causal relationship (34). A clinical study by Xierzhati Aizezi et al. (35). found a significant link between kidney stones and urinary leukocytes (P < 0.05). In our study, elevated urinary leukocytes emerged as an independent risk factor for kidney stone development in hyperuricemic patients, with an associated odds ratio of 1.71 compared to normal levels (P < 0.001). Research has demonstrated a significant positive correlation between elevated serum uric acid levels and new-onset chronic kidney disease (OR, 1.15; 95% CI, 1.05–1.25) (36). Extensive research indicates that elevated serum uric acid may increase oxidative stress, leading to mitochondrial dysfunction, excessive proinflammatory cytokine secretion, and vascular smooth muscle cell proliferation. The potential for tubular injury due to inflammation, mediated by direct physical mechanisms, has been demonstrated in the context of uric acid crystals (37–39). Despite the absence of prior research identifying hematuria as a risk factor for kidney stones in hyperuricemic patients, the present study reveals that the risk of hematuria in hyperuricemic patients is 1.57 times higher than in those without hematuria (P < 0.001). This phenomenon may be attributed to prolonged exposure to elevated serum uric acid levels in patients with hyperuricemia, which can trigger nephritis and fibrosis. These processes subsequently compromise glomerular structure and induce hematuria. It has been demonstrated by several studies that molecules such as nuclein, heat shock protein 90, and membrane-associated protein II, which are released from degraded red blood cells, act as promoters of stone formation (23). Further research indicates that uric acid-induced renal injury instigates immune responses in the renal tubules or surrounding tissues, resulting in the release of pro-inflammatory cytokines. The interaction between these molecules and urate crystals in urine is considered a key mechanism driving urinary stone formation (23, 40, 41).

In this study, we analyzed data from physical examinations of hyperuricemic individuals, revealing that the sole protective factor against kidney stones in this population is meeting daily water intake standards (42, 43). This finding is consistent with prior research indicating that reduced daily water intake is a significant risk factor for kidney stone disease, as evidenced by large cross-sectional studies. Our results align with the known pathomechanism whereby hyperuricemia predisposes individuals to kidney stones. Specifically, increasing daily water intake dilutes serum uric acid and various ions, mitigating renal unit damage and stone formation (4, 23, 43). Restricted cubic spline analysis of age, BMI, and serum uric acid indicated these factors substantially impact kidney stone risk, particularly in diabetic, hypertensive, and male populations. The study emphasizes the need to focus on middle-aged and elderly males aged 40–70 with a BMI exceeding 26.01 kg/m² and serum uric acid levels above 482 µmol/L. Further analysis of age revealed a non-linear relationship with kidney stone risk, with a threshold effect observed. Notably, individuals under 44 years old experienced a statistically significant 6.3% annual increase in kidney stone risk (P < 0.05).

Hypercalciuria has been confirmed as an independent risk factor for kidney stone formation (8, 30). A cross-sectional study found that in patients with Randall plaque-type kidney stones, the urinary calcium and magnesium excretion levels in the NaUr-negative RP group were significantly higher than those in the NaUr-positive RP group (P = 0.02). However, there was no statistically significant difference in urinary uric acid excretion or urine pH between the two groups (31). These findings provide indirect evidence to suggest that the formation mechanism of uric acid-type Randall plaques is unrelated to uric acid concentration in urine and may be driven by serum uric acid concentration. This finding is further substantiated by the findings of a prospective cohort study (44). Consequently, the concentration of calcium ions within the body appears to be a more significant factor than the concentration of uric acid in urine in the formation of kidney stones. Research has found a positive correlation between serum calcium levels and the risk of kidney stone formation. Specifically, for every unit increase in serum calcium, the risk of kidney stone formation increases by 59% (32). Ferraro et al. found that urinary calcium levels are linearly positively correlated with the risk of kidney stone formation, with each 50 mg/d increase in urinary calcium leading to a 31% increase in the likelihood of kidney stone formation (44). Overall, existing research indicates that uric acid concentration in urine has not been found to be associated with kidney stone formation in various clinical studies, but high concentrations of calcium ions in the body are positively correlated with the occurrence of kidney stones.

This study has several limitations. Firstly, although it involved hyperuricemic patients from a multicentre medical examination population, the sample may not be representative of all regions in southern China, limiting generalisability. Secondly, as a retrospective study, it is possible that other potential confounding factors not included in the analysis could influence the results, and therefore causal relationships between risk factors and outcome measures cannot be established. The data for this study were obtained from health examination centres, where routine physical examinations typically do not include metabolic indicators such as serum calcium, urinary calcium, and urinary uric acid. Furthermore, the incorporation of specific disease diagnoses may not strictly adhere to established diagnostic guidelines, which could potentially result in covariate classification bias, thereby significantly impacting the analysis results. These limitations have to some extent restricted the comprehensiveness of the study results. Accordingly, we will launch a multicenter prospective cohort that captures detailed medication histories and prior stone episodes, while expanding the metabolic panel to include calcium, oxalate, citrate, urinary uric acid, parathyroid hormone, and 1,25-(OH)_2_-vitamin D_3_. These analyses will be triangulated with Mendelian randomization to probe potential causal pathways.

## Conclusions

In summary, the prevalence of kidney stones is significantly elevated among hyperuricemic patients in the physical examination population in southern China. Notably, hyperuricemia is more prevalent in men than in women. Obesity, urinary tract infections, and hematuria also emerged as independent risk factors for kidney stones in this population. These findings provide a scientific basis for medical professionals to actively intervene and offer lifestyle guidance to high-risk individuals under 44 with comorbidities such as obesity, diabetes, and hypertension. Such interventions aim to reduce disease risk and enhance quality of life.

## Data Availability

The raw data supporting the conclusions of this article will be made available by the authors, without undue reservation.
