# Supplementary material for: Association between hyperuricemia and kidney stones in Southern China: a multicentre cross-sectional study
Source: Front Endocrinol (Lausanne). 2026 Jan 23;17:1611287. doi: 10.3389/fendo.2026.1611287 (PMC12875968; doi:10.3389/fendo.2026.1611287)

**Supplementary materials**

**Table1S. Assessment of multicollinearity: Variance inflation factors (VIF) of the independent risk factors.**

| **Variables** | **Tolerance** | **VIF** |
| --- | --- | --- |
| Age | 0.953 | 1.049 |
| BMI | 0.977 | 1.023 |
| Sex(male/female) | 0.806 | 1.241 |
| Uric acid | 0.841 | 1.189 |
| Urine WBC counts(+/-) | 0.889 | 1.124 |
| Urine RBC counts(+/-) | 0.931 | 1.074 |
| Water intake | 0.995 | 1.005 |

VIF values were calculated from the final model containing all seven independent variables (Age, BMI, Sex, Uric acid, Urine WBC counts, Urine RBC counts, Water intake).

A VIF value > 5 is often considered indicative of significant multicollinearity. Collinearity diagnostics showed variance inflation factors between 1.005 to 1.241, well below the conventional threshold of 5, indicating no significant multicollinearity among the independent variables.

**Table 2S. Multivariable logistic regression for kidney stones in subgroups stratified by hypertension and diabetes status.**

| **Variables** | **Multivariable** | | |
| --- | --- | --- | --- |
|  | **OR** | **95%CI** | **P-value** |
| **Hypertension (N=1029)** |  |  |  |
| Age | 1.005 | 0.991-1.019 | 0.496 |
| BMI(kg/m^2^) | 1.050 | 1.009-1.094 | **0.017** |
| Sex(male/female) | 2.432 | 1.432-4.130 | **0.001** |
| Uric acid | 1.003 | 1.001-1.006 | **0.002** |
| Urine WBC counts(+/-) | 1.255 | 0.895-1.760 | 0.188 |
| Urine RBC counts (+/-) | 1.728 | 1.271-2.350 | **<0.001** |
| Water intake(Yes/No) | 0.788 | 0.579-1.072 | 0.129 |
| eGFR | 0.996 | 0.986-1.005 | 0.384 |
| **Non-Hypertension (N=1710)** |  |  |  |
| Age | 1.024 | 1.011-1.038 | **<0.001** |
| BMI(kg/m^2^) | 1.082 | 1.042-1.124 | **<0.001** |
| Sex(male/female) | 6.873 | 3.722-12.693 | **<0.001** |
| Uric acid | 1.002 | 1.001-1.004 | **0.017** |
| Urine WBC counts(+/-) | 2.377 | 1.716-3.293 | **<0.001** |
| Urine RBC counts (+/-) | 1.346 | 0.979-1.849 | 0.067 |
| Water intake(Yes/No) | 0.737 | 0.558-0.973 | **0.031** |
| eGFR | 0.995 | 0.985-1.004 | 0.267 |
| **Diabetes mellitus (N=402)** |  |  |  |
| Age | 0.999 | 0.997-1.022 | 0.917 |
| BMI(kg/m^2^) | 1.006 | 0.938-1.078 | 0.871 |
| Sex(male/female) | 3.029 | 1.366-6.718 | **0.006** |
| Uric acid | 1.002 | 0.999-1.006 | 0.188 |
| Urine WBC counts(+/-) | 1.082 | 0.648-1.808 | 0.764 |
| Urine RBC counts (+/-) | 1.818 | 1.100-3.003 | **0.020** |
| Water intake(Yes/No) | 0.787 | 0.477-1.300 | 0.350 |
| eGFR | 0.991 | 0.977-1.005 | 0.191 |
| **Non-Diabetes mellitus (N=2337)** |  |  |  |
| Age | 1.020 | 1.010-1.031 | **<0.001** |
| BMI(kg/m^2^) | 1.084 | 1.053-1.117 | **<0.001** |
| Sex(male/female) | 4.461 | 2.831-7.030 | **<0.001** |
| Uric acid | 1.003 | 1.001-1.004 | **<0.001** |
| Urine WBC counts(+/-) | 1.873 | 1.438-2.440 | **<0.001** |
| Urine RBC counts (+/-) | 1.533 | 1.200-1.958 | **0.001** |
| Water intake(Yes/No) | 0.755 | 0.602-0.946 | **0.015** |
| eGFR | 0.996 | 0.989-1.004 | 0.342 |
| **Hypertension or Diabetes mellitus (N=1177)** |  |  |  |
| Age | 1.006 | 0.993-1.020 | 0.337 |
| BMI(kg/m^2^) | 1.043 | 1.003-1.084 | 0.033 |
| Sex(male/female) | 2.803 | 1.691-4.646 | **<0.001** |
| Uric acid | 1.004 | 1.002-1.006 | **<0.001** |
| Urine WBC counts(+/-) | 1.311 | 0.957-1.796 | 0.092 |
| Urine RBC counts (+/-) | 1.684 | 1.258-2.254 | **<0.001** |
| Water intake(Yes/No) | 0.753 | 0.563-1.008 | 0.057 |
| eGFR | 0.996 | 0.987-1.005 | 0.427 |
| **Non-Hypertension or Non-Diabetes mellitus (N=1562)** |  |  |  |
| Age | 1.023 | 1.009-1.038 | **0.001** |
| BMI(kg/m^2^) | 1.093 | 1.050-1.138 | **<0.001** |
| Sex(male/female) | 6.342 | 3.336-12.055 | **<0.001** |
| Uric acid | 1.002 | 0.999-1.004 | 0.073 |
| Urine WBC counts(+/-) | 2.417 | 1.703-3.430 | **<0.001** |
| Urine RBC counts (+/-) | 1.391 | 0.994-1.946 | 0.054 |
| Water intake(Yes/No) | 0.768 | 0.574-1.029 | 0.077 |
| eGFR | 0.994 | 0.983-1.004 | 0.221 |

Note: This sensitivity analysis was conducted to indirectly assess potential confounding by unmeasured medication use (e.g., antihypertensive drugs such as diuretics), by examining the consistency of the association between serum uric acid and kidney stones across population subgroups with different probabilities of medication exposure.

Adjusted variables: All models within each stratum were adjusted for the same set of covariates: age, sex, BMI, serum uric acid, urine WBC counts, urine RBC counts, water intake, and eGFR.

**Table 3S. Multivariate Logistic Regression Analysis of Risk Factors for Kidney Stones in Different CKD Stage Subgroups**

|  | **CKD G1 Stage**  **(N=2008)** | **CKD G2 Stage**  **(N=674)** | **CKD G3-4 Stage**  **(N=57)** | **P for interaction** |
| --- | --- | --- | --- | --- |
|  | **OR (95% CI)** | **OR (95% CI)** | **OR (95% CI)** |  |
| Age | 1.024 (1.013-1.034) | 1.010 (0.996-1.025) | 1.030 (0.976-1.088) | 0.293 |
| BMI(kg/m^2^) | 1.088 (1.055-1.122) | 1.055 (0.994-1.119) | 1.021 (0.856-1.217) | 0.316 |
| Sex(male/female) | 3.618 (2.299-5.695) | 6.510 (2.759-15.358) | 3.637 (0.457-28.921) | 0.371 |
| Uric acid | 1.002 (1.001-1.004) | 1.005 (1.002-1.007) | 1.003 (0.996-1.010) | 0.207 |
| Urine WBC counts(+/-) | 1.380 (1.029-1.850) | 2.654 (1.719-4.097) | 2.790 (0.808-9.632) | 0.075 |
| Urine RBC counts (+/-) | 1.741 (1.336-2.268) | 1.414 (0.931-2.146) | 0.626 (0.174-2.251) | 0.202 |
| Water intake(Yes/No) | 0.795 (0.622-1.017) | 0.710 (0.478-1.054) | 0.578 (0.122-2.734) | 0.505 |

Adjustment variables include: age, sex, BMI, serum uric acid, Urine WBC counts, Urine RBC counts and water intake.

**Supplementary Figure 1**


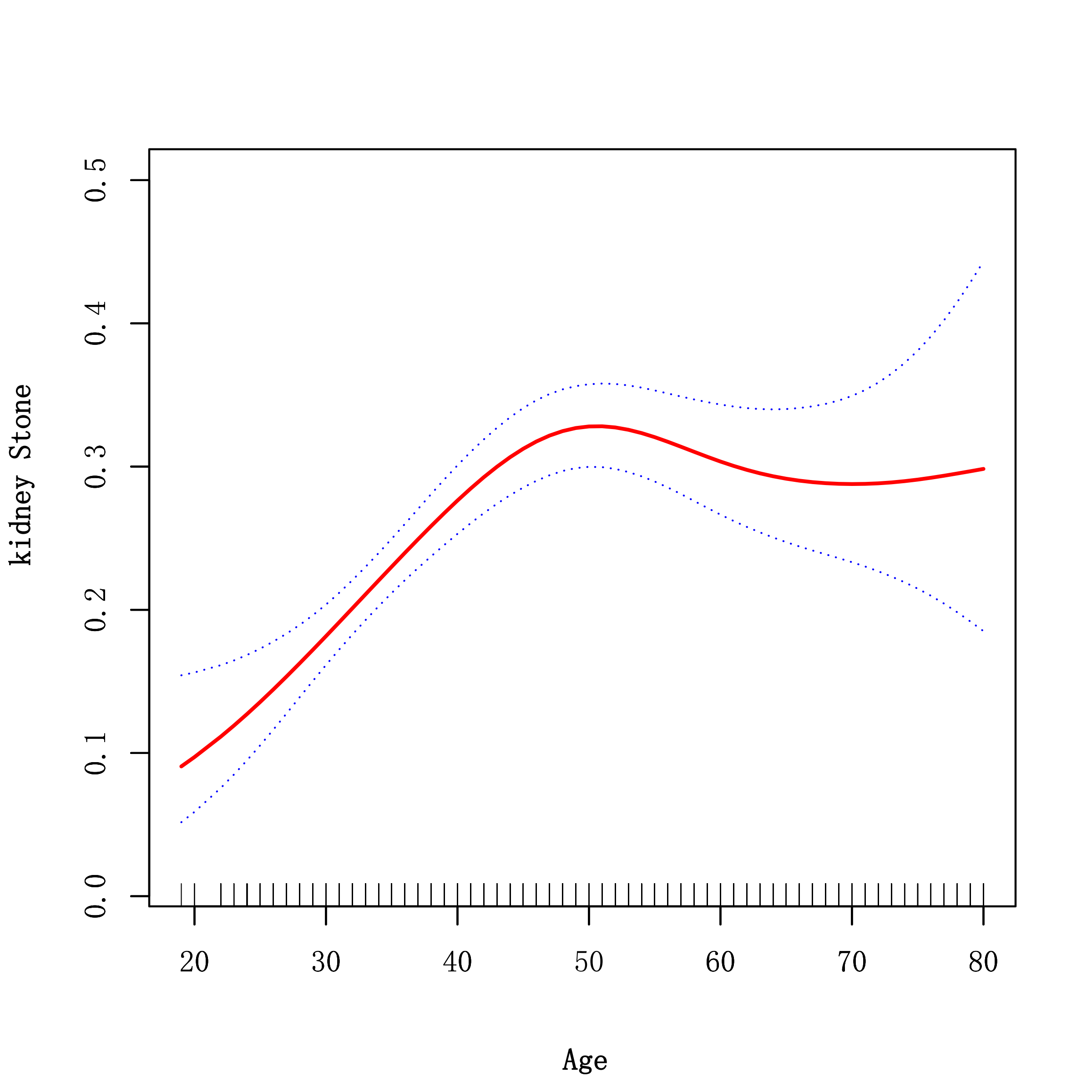


**Ethical approval documents**

1. Luzhou Maternal and Child Health Hospital (Luzhou Second People's Hospital)


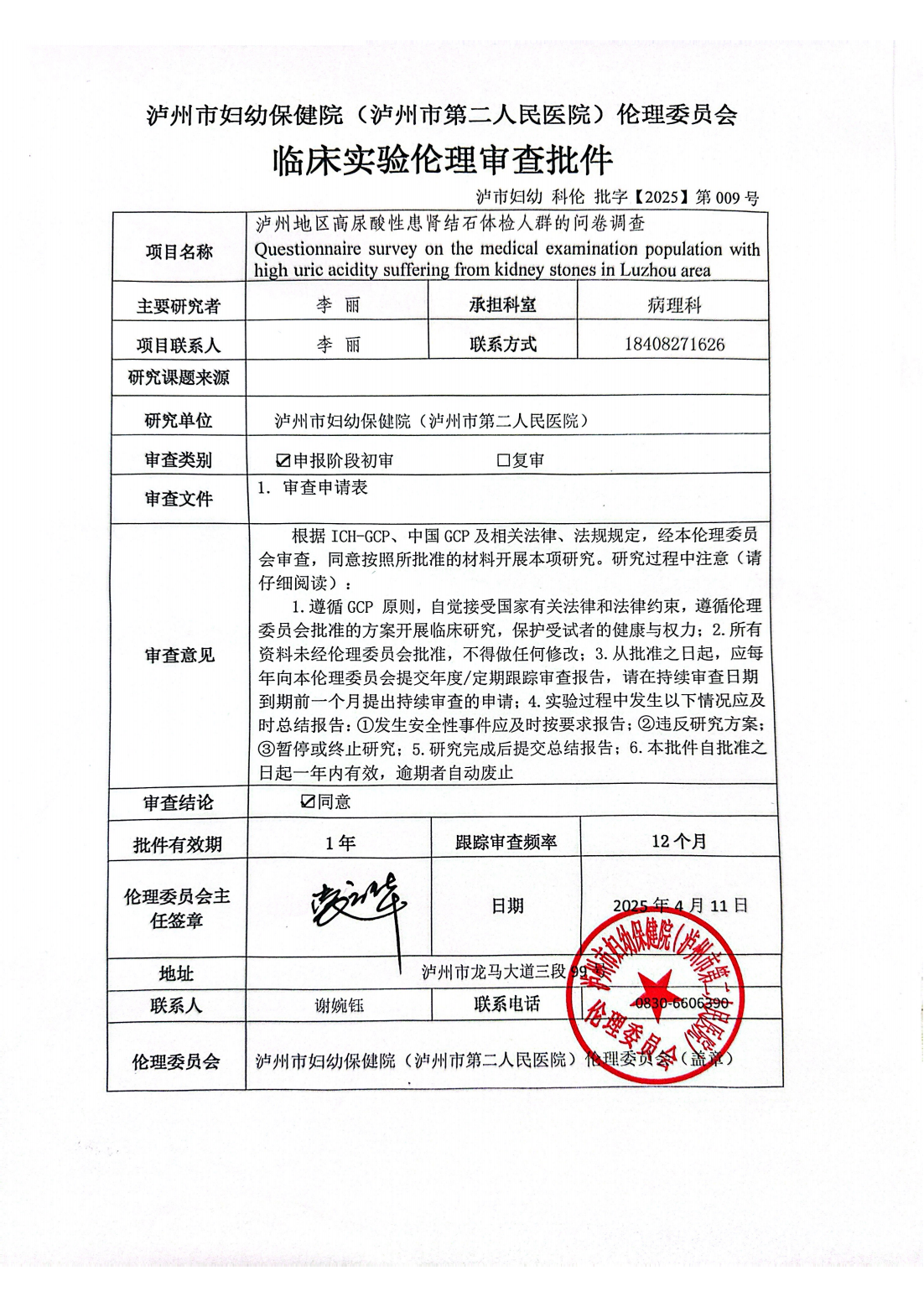


1. Sichuan Province, the Traditional Chinese Medicine Hospital of Southwest Medical University


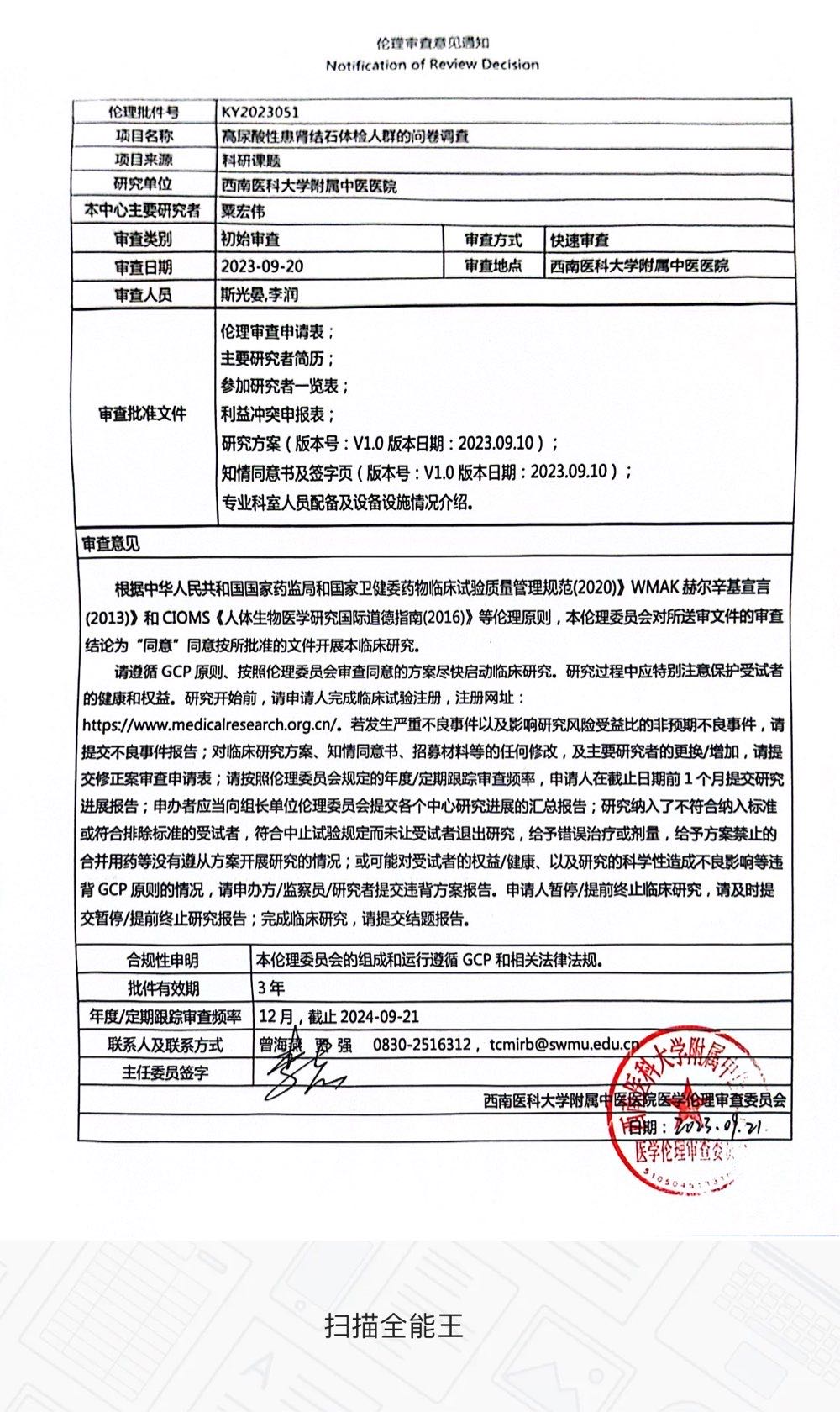


1. the Ethnic Hospital of Guangxi Zhuang Autonomous Region


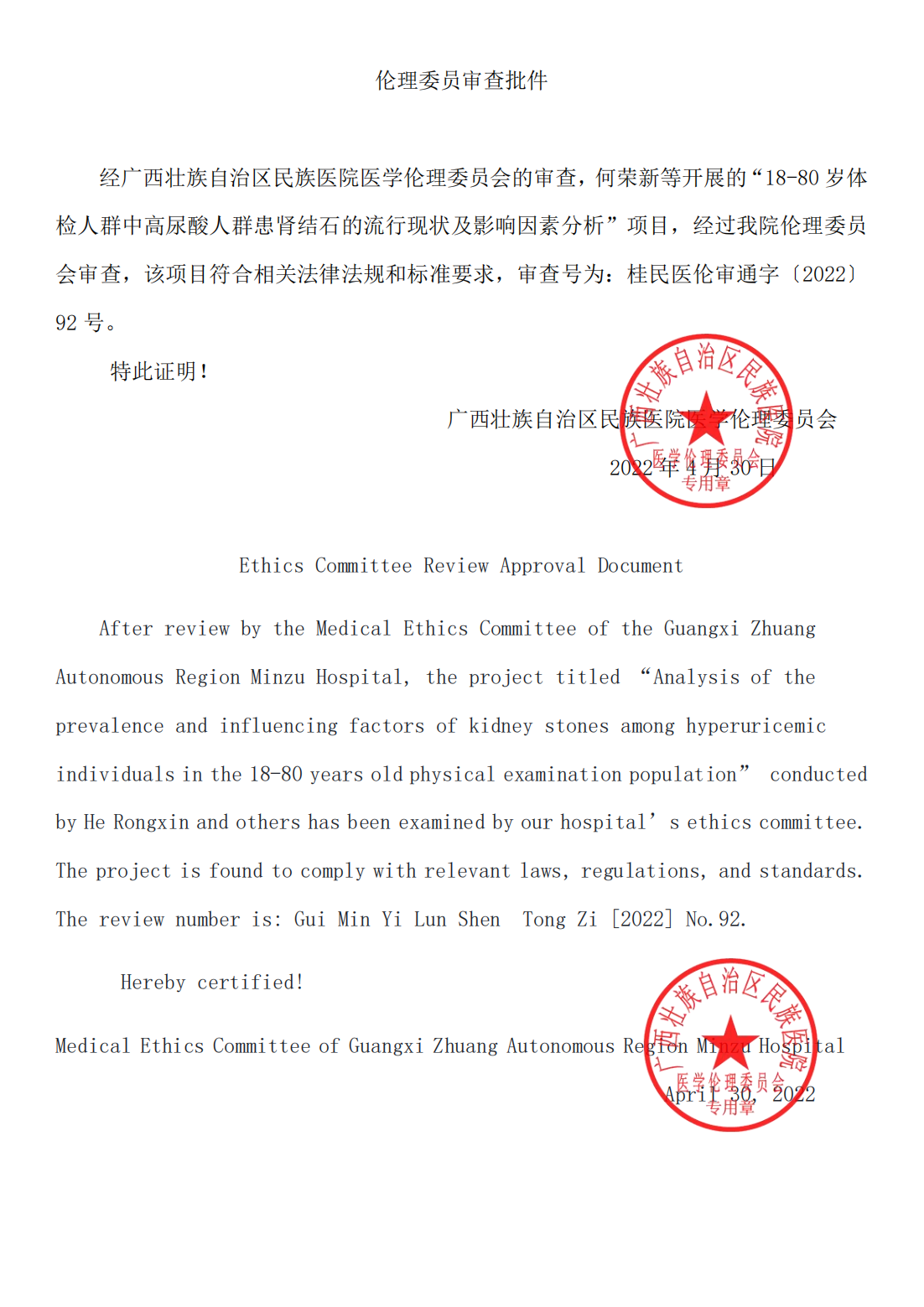


1. the People's Hospital of Ganxian County, Ganzhou City, Jiangxi Province


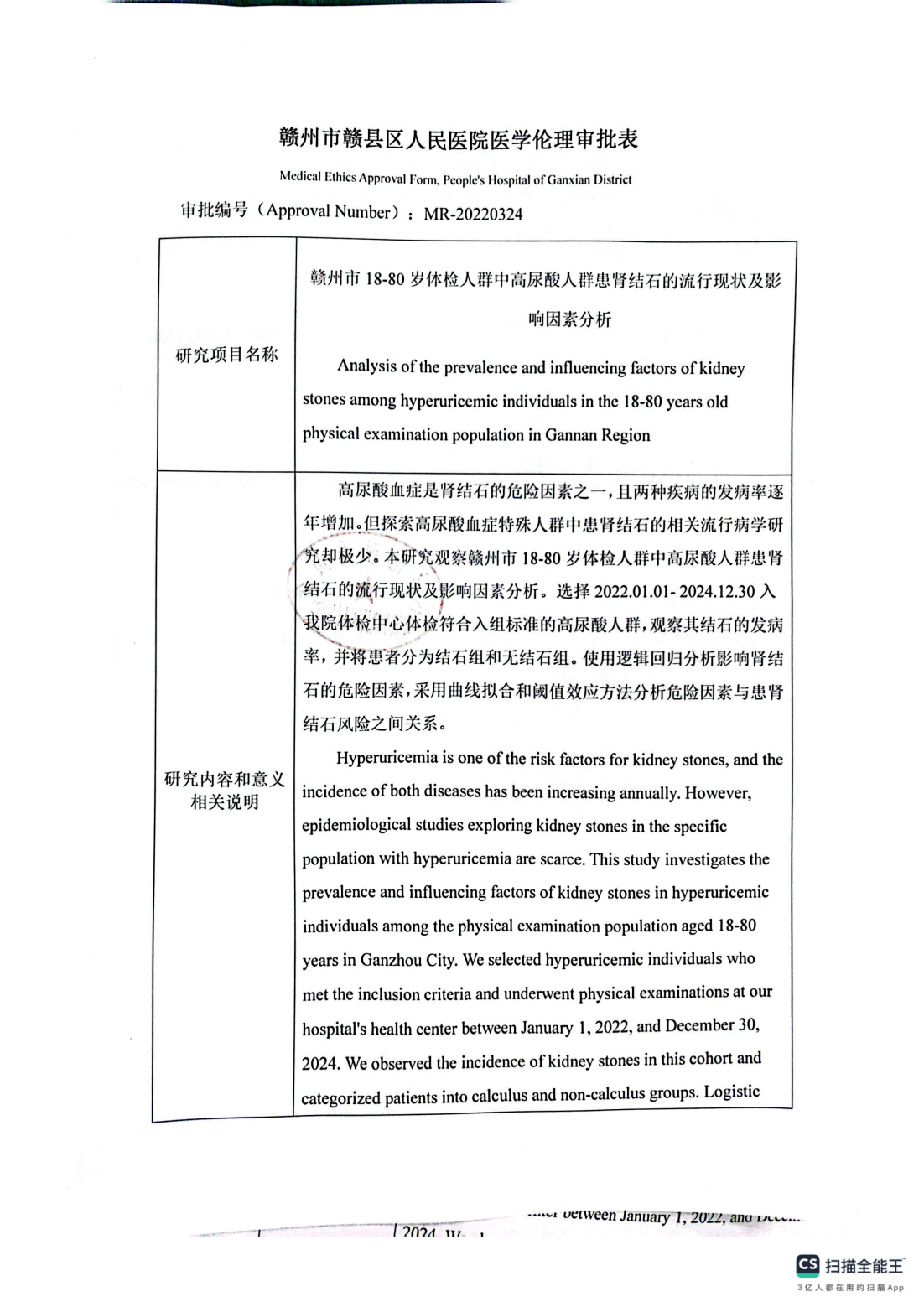

Supplement: Supplementary file 1 [file DataSheet1.docx]
